# Association of Combined Enzymatic and Surgical Debridement with Clinical Outcomes in Extensive Burn Patients

**DOI:** 10.3390/jcm14155233

**Published:** 2025-07-24

**Authors:** Yasuhiko Kaita, Mikio Nakajima, Takeaki Matsuda, Yoshihiro Yamaguchi

**Affiliations:** 1 Department of Trauma and Critical Care Medicine, Kyorin University School of Medicine, Tokyo 1818611, Japan; 2 Emergency and Critical Care Center, Tokyo Metropolitan Hiroo Hospital, Tokyo 1500013, Japan

**Keywords:** NexoBrid^®^, excision, infection control

## Abstract

**Background/Objectives**: Burned tissue has traditionally been removed surgically, but the effectiveness of enzymatic debridement with NexoBrid has been reported in burn patients and has gained attention in recent years. This agent was approved for use in Japan in 2023. However, even in Japan, there have been few studies examining its effectiveness in patients with extensive burns. The purpose of this study was to analyze the association of combined NexoBrid and surgical excision with clinical outcomes in extensive burn patients. **Methods**: Between January 2020 and December 2024, seventeen flame burn patients requiring surgical excision were divided into two groups based on whether NexoBrid was used. Clinical outcomes between the two groups were compared using the propensity score overlap weighting method to adjust for baseline differences. **Results**: Seven of the patients received combined NexoBrid and surgical excision. After weighting, NexoBrid was significantly associated with a shorter time to complete debridement of burned tissue (difference −4 days, 95% CI −5 to −2) and lower percentage of bacteremia (odds ratio 0.06, 95% CI 0.00 to 0.96). However, no significant differences were observed in the length of ICU stay, the amount of blood transfusion required for complete tissue removal, hospitalization costs, and in-hospital mortality. **Conclusions**: Combining conventional surgical excision with enzymatic debridement may reduce the time required to complete debridement of burned tissue and decrease the rate of bacteremia. Further studies are needed to evaluate the effectiveness of NexoBrid combined with surgical excision in patients with extensive burns.

## 1. Introduction

Burns are one of the most serious injuries, and approximately 11 million burn patients worldwide require medical treatment each year [1]. Approximately 180,000 deaths every year are caused by burns [2]. Despite recent advances in burn care, severe burns can be fatal, and non-fatal burns can lead to morbidity. According to a report from the Japanese Society for Burn Injury, approximately 1500 patients require acute inpatient hospitalization annually, of which around 150 die [3]. In Tokyo, more than 300 severe burn patients are treated in the burn units each year, and the mortality rate shows a mildly decreasing trend [4]. Previous studies have reported that most deaths of burn patients were due to infections, including in Japan [4,5]. Therefore, infection control is integral for the outcomes of burn patients.

The debridement of burn tissue is crucial for controlling mortality and infections [6,7,8,9]. The gold standard treatment for burn patients is surgical debridement followed by skin grafting [10]. Although there have been advances in burn wound care, wound management remains challenging.

Over the years, various products have been studied for effective debridement, but their application in clinical practice has been limited due to low efficacy or significant side effects. In the late 19th century, bromelain, a proteolytic enzyme extracted from the stems of pineapples, began to attract attention, with reports indicating its ability to promote wound healing by selectively dissolving necrotic tissue. In 2012, NexoBrid (MediWound Ltd., Yavne, Israel) was introduced to the European market following promising outcomes in several reports. NexoBrid is a concentrated formulation of proteolytic enzymes rich in bromelain. NexoBrid is indicated for the removal of eschar in burn patients with deep partial and full-thickness burns. Each bottle contains 5 g of NexoBrid and is applied to a burn wound area covering 2.5% of the total body surface area. The price of one bottle is 162,995.5 yen in Japan.

The effectiveness of NexoBrid has been reported to reduce the need for and extent of surgical debridement, reduce blood loss, and improve functional and cosmetic outcomes compared with surgery [11,12,13,14,15,16]. Moreover, much of the previous literature on enzymatic debridement of burn wounds has mentioned its use in smaller burns and specialized sites such as the hands, feet, and genitalia [14,15,17], but there are few papers on the significance of NexoBrid for extensive burns.

NexoBrid was approved in Japan in 2023, and several case reports have described its effectiveness in hand and foot burns [18,19]. However, even in Japan, few studies have investigated its effectiveness in patients with extensive burns. This pilot study aimed to evaluate whether the combination of enzymatic debridement using NexoBrid and surgical excision is associated with improved clinical outcomes in patients with extensive burns.

## 2. Materials and Methods

### 2.1. Patient Selection

This single-center retrospective cohort study was conducted at Kyorin University Hospital (Tokyo, Japan). We included consecutive patients with extensive burns who underwent surgical excision from January 2020 to December 2024. Extensive burn was defined as burns involving 30% or more of the total burn surface area (TBSA), based on previous reports [3,4]. Because the mechanism of burn injury influences treatment strategy, the study included only patients with flame burns. Patients with isolated inhalation injury, those in cardiac arrest, and those with burns covering 30% of the TBSA or less were excluded.

Typically, patients with flame burns are promptly considered for surgical excision due to the depth of injury upon admission. The decision for surgical excision was made by burn surgeons based on the depth and location of the burn wound, and patients who did not undergo surgical excision were excluded. Thus, the included patients were those transported to our burn center during the study period with flame burns covering 30% or more of the TBSA who underwent surgical excision.

Patients were divided into those treated with NexoBrid (NexoBrid group) and those treated without NexoBrid (non-NexoBrid group). Since the post-debridement treatment strategy remained unchanged after 2020, the study period was set to begin in 2020. In Japan, NexoBrid became available in August 2023. NexoBrid can be used for deep partial-thickness burns and full-thickness burns in Japan. Burn surgeons decided to apply NexoBrid based on the depth and location of the burn wound. Although NexoBrid was used for both flame and scald burns, only patients with flame burns were included in this study to standardize the injury mechanism. From the time of its approval until December 2024, NexoBrid was used for all flame burn patients with burns covering 30% or more of the TBSA who were transported to our burn center. Therefore, the study period ended in December 2024. In summary, the NexoBrid group included patients who received enzymatic debridement with NexoBrid and surgical excision after the drug’s approval, while the non-NexoBrid group included patients who underwent conventional surgical excision prior to NexoBrid availability.

The following data were collected from medical records: age, sex, the percentage of TBSA, the percentage of full-thickness burn area (FTBA), the burn index, the prognostic burn index, the presence of inhalation injury, intensive care unit (ICU) length of stay, the timing of debridement completion, the amount of blood transfused to complete the removal of burned tissue, hospitalization costs, presence of bacteremia, and in-hospital mortality.

The primary outcomes of this study were in-hospital mortality and the presence of bacteremia. The secondary outcomes included ICU length of stay, timing of debridement completion, the amount of blood transfused to complete the removal of burned tissue, and hospitalization costs.

### 2.2. Enzymatic Debridement

Burn specialists evaluated all burn patients on admission to determine suitability for enzymatic debridement. Before applying NexoBrid, we cleansed the wound, thoroughly removed blisters, and routinely pre-soaked the burn wound with saline-soaked gauze for 2 h. However, in cases admitted shortly after an injury, pre-soaking was not performed. NexoBrid was applied to the burn wound, covered with polyurethane film, and left for 4 h. The treatment area was debrided to remove dissolved tissue and drug residues, then soaked with saline-soaked gauze for two hours. The burn wound was then covered with a generous amount of Vaseline and non-adherent gauze to keep wounds moist. All patients received ketamine for analgesia during the application and removal of NexoBrid. Skin grafting was recommended two days after removal of the Nexobrid, and autologous skin grafting was performed depending on the condition of the burn wound.

### 2.3. Surgical Debridement

Before the approval of NexoBrid, burn wounds were surgically excised according to their depth. If the dermis could be preserved, an autologous skin grafting was applied, and the wounds were covered with an artificial dermis if fascial excision was performed.

### 2.4. Skin Grafting

After preparing the wound bed, the split-thickness skin grafting was performed using the MEEK^®^ micrograft cutting device (Humeca, Borne, The Netherlands). Additionally, the autologous skin cell suspension (RECELL^®^, Avita Medical, Inc., Valencia, CA, USA, Rigenera^®^ Human Brain Wave, Turin, Italy) was used with the skin graft to achieve an earlier wound closure in all cases.

### 2.5. Wound Management

As a general rule, the wounds were washed daily with saline and applied with ointment containing bacitracin and fradiomycin sulfate. The basic fibroblast growth factor was also sprayed daily in preparation for the skin grafting.

### 2.6. Stastical Analysis

Continuous variables were expressed as medians with interquartile ranges, and binary variables were expressed as numbers with percentages. Continuous variables were compared using the Mann–Whitney U test, and binary variables were compared using Fisher’s exact test.

To adjust baseline difference between the NexoBrid and non-NexoBrid groups, the propensity score overlap weighting method was applied [20,21]. The propensity score model included age, sex, inhalation injury, and TBSA. As a sensitivity analysis, generalized linear regression models adjusted by propensity scores were also conducted. In these models, binary outcomes were presented as odds ratios, and continuous outcomes were presented as mean differences, both with 95% robust confidence intervals. Two-sided *p*-values of <0.05 were considered significant. All statistical analyses were performed using Stata MP19 (StataCorp, College Station, TX, USA).

## 3. Results

### 3.1. Included Patients

**Figure 1:**
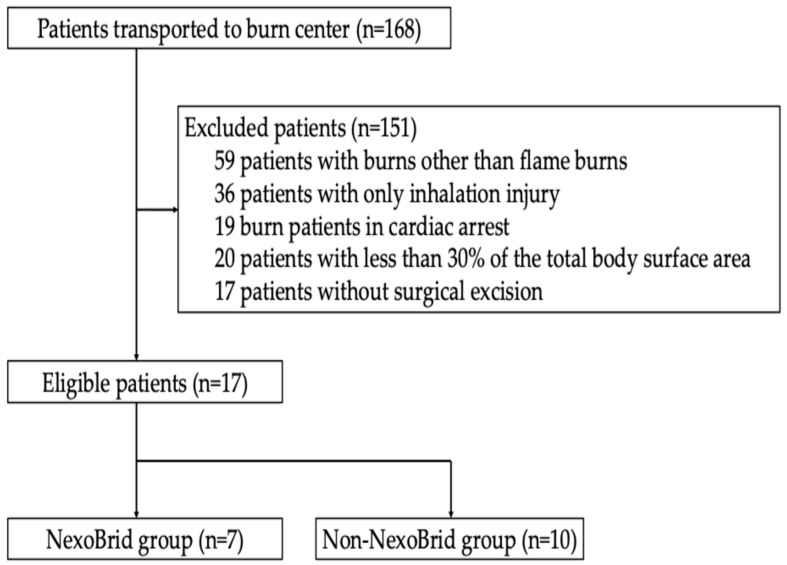
Patient flow of the present study.

During the 5-year study period, 168 burn patients were admitted to Kyorin University Hospital. After applying the exclusion criteria, 17 burn patients were included in the current study (Figure 1).

### 3.2. Background of Burn Patients

Enzymatic debridement using NexoBrid was performed on seven patients, and surgical debridement alone was performed on ten patients. Table 1 presents the background characteristics of the enrolled patients. There were no significant differences between the two groups in terms of age, sex, presence of inhalation injury, TBSA, FTBA, or burn index.

### 3.3. Outcomes of the Enrolled Patients

Table 2 displays unadjusted outcomes of the enrolled patients. There was no significant difference in in-hospital mortality between the two groups. The incidence of bacteremia was lower in the NexoBrid group compared to the non-NexoBrid group. However, this difference was not statistically significant. No significant differences were observed between the two groups regarding ICU length of stay or the amount of blood transfusion needed for debriding burned tissue. The time required to complete debridement after injury was significantly shorter in the NexoBrid group. Although hospitalization costs tended to be higher in the NexoBrid group, the difference was not statistically significant.

### 3.4. Association of Combined Enzymatic and Surgical Debridement with Outcomes

Table 3 presents outcomes after propensity score overlapping weighting. After weighting, the use of NexoBrid was not significantly associated with mortality, though it was associated with the presence of bacteremia. There were no significant differences in ICU length of stay, transfusion volume, or hospital costs, though there was a significant difference in time to complete total debridement. The results of the sensitivity analysis using propensity score adjustment were similar to the results of propensity score overlap weighting.

### 3.5. Details of Patients with Enzymatic Debridement

Table 4 presents details about the patients treated with NexoBrid. In six of the seven cases, NexoBrid was used on the day of injury, while in the remaining case, it was applied the day after. In two instances, it was used shortly after the injury without pre-soaking. The area of the wound using NexoBrid was 30% in six cases and 15% in one case. All burn wounds except the area where NexoBrid was applied were surgically removed. Applied sites included the face in three cases, the neck in three, the upper limbs in six, the hands in one, the trunk in four, the lower limbs in four, and the foot in one. The bacteria detected in the blood were *Pseudomonas aeruginosa*, *Stenotrophomonas maltophilia*, *Candida* species. There were two fatal cases, both of which occurred due to wound infection more than one month after burn injury.

### 3.6. Case 4

**Figure 2:**
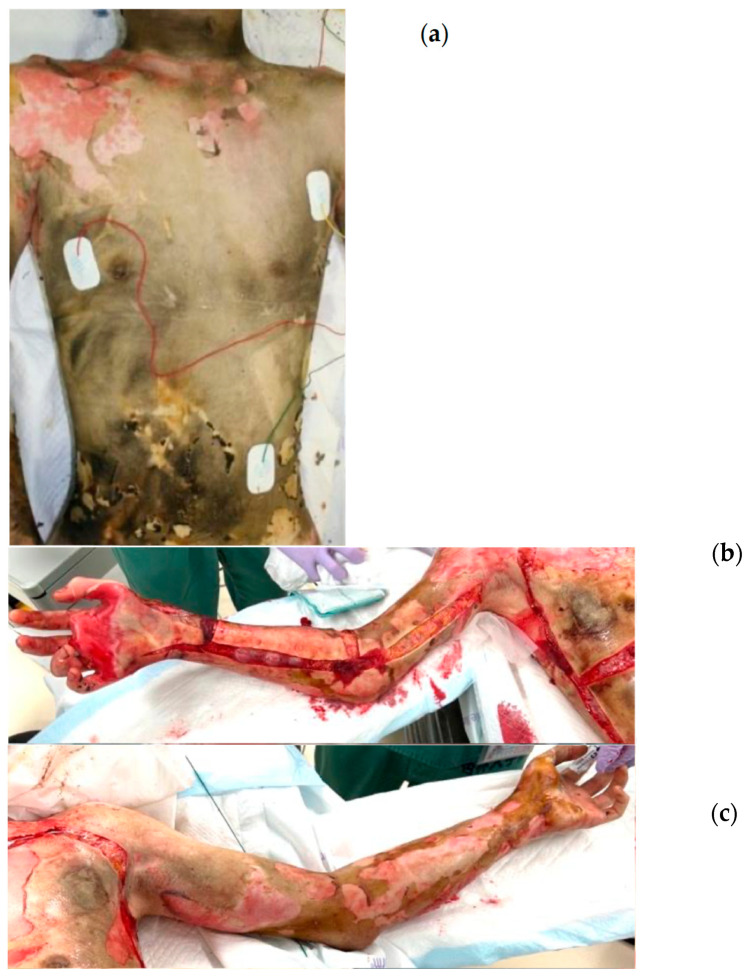

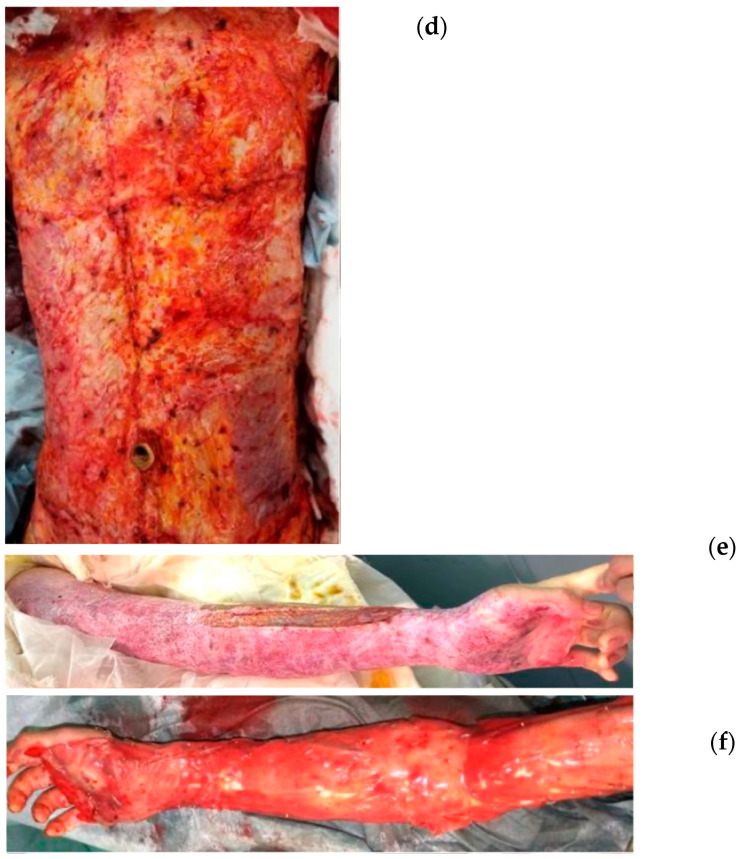
Case 4. (**a**) The chest and abdomen on admission. (**b**) Findings of the right limb after an escharotomy on the day of the injury. (**c**) Findings of the left limb after an escharotomy on the day of the injury. (**d**) Findings of the chest and abdomen following a fascial excision on the day of the injury. (**e**) Findings of the left limb after an enzymatic debridement on the day of the injury. (**f**) The right limb was covered with an artificial dermis after the fascial excision on post-burn day 1.

A 21-year-old male patient suffered self-inflicted burns and was transported to our burn center. He sustained 50% TBSA burns to the neck, trunk, both upper limbs, and left thigh, accompanied by inhalation injury (Figure 2a–c). The burn depth was diagnosed as clinically deep, and an escharotomy was performed on both upper limbs (Figure 2b,c). On the day of the injury, a fascial excision was carried out on the burned area of the trunk (Figure 2d), with pre-soaking applied during the surgery. NexoBrid was applied to the left upper limb and left thigh after the operation (Figure 2e). The day following the injury, the remaining burn wounds on the right upper extremity and back were surgically excised, and all burn tissue was completely removed. The surgically excised areas were covered with artificial dermis (Figure 2f), and skin grafting was performed multiple times, starting two weeks after the injury. On the other hand, skin grafting was conducted three weeks after the enzymatic removal of the affected areas, and all wounds were closed three months post-injury.

### 3.7. Case 5

**Figure 3:**
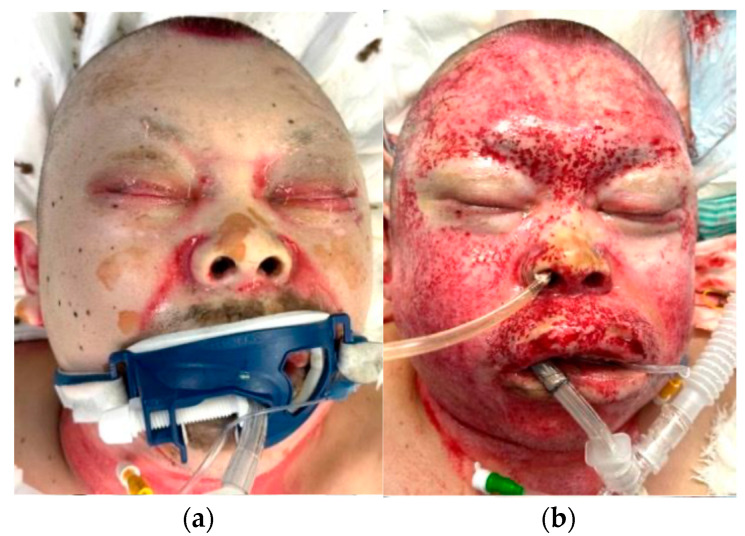
Case 5. (**a**) The face on admission. (**b**) Findings after an enzymatic debridement on the day of injury.

A 60-year-old man suffered flame burns in a house fire. He sustained 43% TBSA burns to the head, face, neck, trunk, both hands, and both lower limbs, accompanied by inhalation injury (Figure 3a). The burn depth was diagnosed to be a mixture of superficial and deep dermal burns. Enzymatic debridement was performed on the face, neck, and both hands immediately without pre-soaking (Figure 3b). The intratissue pressure on the dorsum of the left hand was 52 mmHg before application of NexoBrid, but it had dropped to 20 mmHg 4 h after applying the drug. Similarly, the intratissue pressure on the dorsum of the right hand had dropped from 36 mmHg to 15 mmHg. As some areas of the necrotic tissue on the face had not been removed effectively, NexoBrid was applied again the following day, and was effective. Four days later, the remaining burn wound was surgically excised, and a skin graft was performed, and all burn tissue was completely removed. After that, skin grafting was repeated, and the wound was closed 2 months after the injury.

### 3.8. Case 6

**Figure 4:**
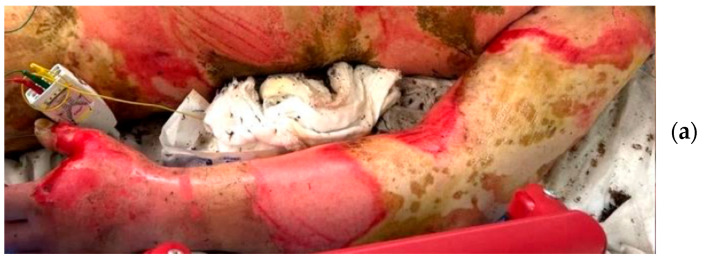

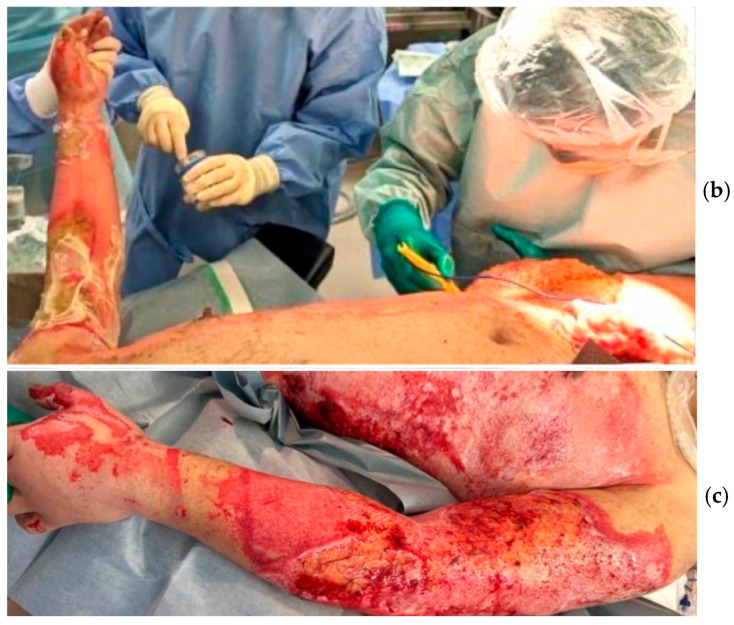
Case 6. (**a**) The left limb on admission. (**b**) Scenes in which surgical excisions and enzymatic debridement are performed simultaneously in the operating room on the day of injury. (**c**) Findings after an enzymatic debridement on the day of injury.

A 54-year-old man suffered flame burns while performing electrical work. He sustained 52% TBSA burns to the trunk, left upper limbs, and both lower limbs (Figure 4a). The burn depth was diagnosed to be a mixture of superficial and deep dermal burns and deep burns. Because the patient was transported to the hospital soon after the injury, we immediately performed fascial excision of the burn wounds on the anterior of both lower limbs in the operating room (Figure 4b). At the same time, we applied NexoBrid to the left upper limb and trunk without pre-soaking (Figure 4c). The day following the injury, the remaining burn wounds on the posterior of both lower limbs were surgically excised, and all burn tissue was completely removed. In addition, when the intratissue pressure of the upper limbs was measured, it was 81 mmHg in the left upper arm and 55 mmHg in the left forearm before application of the drug, but it decreased over time, and 4 h after application it was 12 mmHg in the left upper arm and 5 mmHg in the left forearm. Skin grafting was performed on the enzymatically excised area from the 5th day after injury, and on the surgically excised area from 2 weeks after injury, with the wound finally closed 2 months after injury.

### 3.9. Details of Patients with Only Surgical Debridement

Table 5 presents details of the patients who underwent conventional surgical excision without the use of NexoBrid. The initial debridement was performed within 24 h after burn injury in all cases. There were nine cases of bacteremia, and the bacteria detected in the blood were *Pseudomonas aeruginosa*, *Stenotrophomonas maltophilia*, *Candida* species, and Enterococcus species. There were three fatal cases, all of which occurred one month or more after injury due to wound infection, similar to the deaths in the NexoBrid group.

## 4. Discussion

Most studies on NexoBrid have focused on small or specific areas, such as the hands, face, and genitals, with few conducted on larger areas. Hofmaenner et al. demonstrated that enzymatic debridement using NexoBrid was safe for burns covering more than 15% of the body surface area and did not significantly affect hemodynamics or inflammation [22]. Bowers et al. showed that NexoBrid is more beneficial for patients with extensive burns, as it reduces blood transfusions, preserves the dermis, minimizes the need for skin grafting, and lowers the number of surgeries in patients with an average burned surface area of 30% [16]. Although the number of cases included in this study was small compared to these two reports, the median burn area of patients in this study was more extensive at 50%. This study provided some insight into the association between NexoBrid use and clinical outcomes in patients with extensive burns.

This study indicated an association between the use of NexoBrid and the prevalence of bacteremia. Regarding the link between NexoBrid use and infection, enzymatic debridement with NexoBrid has been reported to slightly reduce infection rates by removing necrotic tissue [23]. Additionally, a previous report showed that patients with extensive burns of 30% or more who underwent surgical excision of all burned tissue within one week had a significantly lower rate of bacteremia [24]. These findings suggest that the difference in bacteremia observed in this study may be due to the use of NexoBrid, which enabled early debridement. Although there was an association with bacteremia, which is linked to a poor prognosis, no difference in mortality was found in the current study. Since Bowers et al. suggested that NexoBrid might be more effective for extensive burns [16], its effectiveness in improving prognosis is expected to increase with more experience. Further research with more cases is needed.

In this study, burn tissue resection was completed earlier in the NexoBrid group than in the non-NexoBrid group. The complete resection of burn tissue was achieved four days earlier in the NexoBrid group compared to the non-NexoBrid group. This is consistent with previous reports [25]. Moreover, the assessment time was earlier than in previous reports, although the burned area in the current study was more extensive. These effects are thought to stem from the convenience of this debridement agent. Surgical debridement requires experience and expertise and can generally only be performed in an operating room. In contrast, NexoBrid is easy to use, highly effective, and facilitates debridement both at the bedside and in the operating room. In fact, enzymatic debridement was performed in the ICU, except for Case 6. Although Cases 3 and 6 involved extensive burns exceeding 50%, the removal of the burned wound could be completed the day after the injury by combining surgical and enzymatic debridement. With conventional surgical excision, only 15 to 20% of the burned tissue can be removed at one time due to time constraints, so even if the surgery is performed daily, it would take more than three days to complete the resection of the burned tissue. In Case 6, surgical excision and enzymatic debridement were conducted simultaneously in the operating room, and the strategy of surgically excising the full-thickness burns while enzymatically debriding the superficial and deep dermal burns may be reasonable. In addition, the anatomical location of burns might influence the completion of the removal of burned tissue. NexoBrid was applied to the face and hands in this study, which are typically difficult to surgically remove. However, as noted in previous reports, it may also be beneficial for treating severe burns. Conducting surgical intervention on areas like the trunk, where it is easier to excise surgically, while performing enzymatic debridement in more challenging regions, is a very rational approach to early removal of burn necrotic tissue.

Pre-soaking was not performed in two of the seven cases in this study, but the efficacy of NexoBrid can be expected without pre-soaking for flame burns in patients who are transported to the hospital early after the injury. Bowers et al. reported that it was effective without pre-soaking for circumferential burns [16], and it is possible that the removal of burned tissue could be performed more quickly if it can be performed without pre-soaking.

In this study, NexoBrid was applied to the limbs, where it had previously shown promise. Three of the seven cases involved circumferential burns on the extremities. In Case 3, an escharotomy was performed, which is the traditional treatment for this type of injury. In Cases 5 and 6, enzymatic debridement was conducted, leading to a decrease in intratissue pressure without surgical intervention. The effectiveness of NexoBrid for circumferential burns aligns with earlier reports. The escharotomy reaches the fatty tissue as noted in Case 3, while enzymatic debridement preserves the normal dermis, potentially benefiting aesthetics and function. However, the effects of NexoBrid are not definitive, making it essential to continuously evaluate intratissue pressure over time.

Regarding the amount of blood transfusion required for the complete removal of burned tissue in this study, the difference was not significant. This may be because the area of enzymatic debridement was large (30%) in six out of seven cases, and the NexoBrid group included cases in which surgical resection was also used. Further investigation is needed, including a comparison of the amount of bleeding by site.

In addition to its convenience, the advantages of this drug include its potential to preserve normal dermis, and it has been reported that enzymatic debridement results in better functional and cosmetic outcomes than surgical debridement [15,22]. Shoham et al. reported that concerns that delayed wound healing contributes to the formation of hypertrophic scars are unfounded. However, the burn area of the patients in this study was less than 30%, and the severity of extensive burns leads to a strong systemic inflammatory response, which is of concern. Evaluation of extensive burns will need to be further investigated. Moreover, functional and cosmetic evaluations have not yet been performed in Japan, and the quality of scars needs to be evaluated in the future.

There have been few reports on the economic evaluation of NexoBrid. Minic et al. reported that hospitalization costs were significantly reduced when comparing a group that underwent conventional surgical debridement with a group that underwent enzymatic debridement using NexoBrid [26]. Although the drug costs were higher in the Nexobrid group, the reduction in blood transfusion volume and the shortened hospital stay led to the reduction in hospitalization costs. In this study, hospitalization costs were higher in the NexoBrid group. One factor contributing to this is that the average burn area in the previous report was 24%, whereas the median burn area in this study was 35%, which was extensive. Additionally, NexoBrid was used over a wider area, potentially leading to higher drug costs. Another factor may be that there was no significant difference in the amount of blood transfusion or the length of stay in the ICU. The usefulness of NexoBrid is more pronounced when the burn area is small, but further case experience is needed for extensive burn patients.

Our study has several limitations that should be acknowledged. First, it was a retrospective analysis conducted at a single center with a small sample size. Larger, prospective, multi-center studies are necessary to determine whether the combination of NexoBrid with surgical excision is statistically significantly better than conventional treatment. Second, the assessment of NexoBrid was incomplete, as long-term outcomes such as scar quality were not evaluated. Third, although burn care team and treatment protocols remained unchanged before and after the introduction of NexoBrid, annual variations were not accounted for in this study. Finally, there was no standardization regarding the timing of NexoBrid application or whether pre-soaking was used. Most importantly, this study should be considered a pilot study with a limited number of cases, and more extensive research is required. This study did not recommend the use of NexoBrid for severe burn patients due to the small sample size and the lack of statistically significant results in outcomes like prognosis. Nevertheless, the results are promising, and NexoBrid could potentially be an effective debridement agent. Therefore, follow-up studies with larger patient populations are needed to confirm these findings.

## 5. Conclusions

Combining traditional surgical excision with enzymatic debridement may reduce the time required to complete debridement of burned tissue and decrease the prevalence of bacteremia. However, further studies are necessary to assess its effectiveness in patients with extensive burns.

## Figures and Tables

**Table 1:** Patient background.

Variables	Total (n = 17)	NexoBrid Group (n = 7)	Non-NexoBrid Group (n = 10)	*p*-Value
Age (years)	59 (50–71)	54 (25–60)	71 (50–79)	0.10
Male, n (%)	13 (76)	5 (71)	8 (80)	>0.99
Inhalation Injury, n (%)	11 (65)	6 (86)	5 (50)	0.30
TBSA (%)	46 (34–55)	50 (46–55)	38 (32–58)	0.12
FTBA (%)	22 (18–28)	32 (9–50)	21 (18–23)	0.30
Burn Index	35 (27–43)	43 (27.5–50)	31.25 (27–39)	0.19

Data were presented as n (%) for binary variables and median (interquartile range) for continuous variables. TBSA: total burn surface area, FTBA: full-thickness burn surface area.

**Table 2:** Patient outcomes.

Variables	All Cases (n = 17)	NexoBrid Group (n = 7)	Non-NexoBrid Group (n = 10)	*p*-Value
In-hospital mortality	5 (29)	2 (29)	3 (30)	>0.99
Bacteremia	14 (82)	5 (71)	9 (90)	>0.99
ICU-LOS (days)	74 (54–114)	77 (65–119)	69 (35–114)	0.46
Total debridement completion, PBD	4 (3–6)	2 (2–4)	6 (4–7)	0.009
RBC transfusion, mL	3080 (1680–3920)	3080 (1680–4480)	3360 (280–3920)	0.84
FFP transfusion, mL	3360 (1200–4800)	3360 (1200–4800)	3600 (1200–5760)	0.96
PC transfusion, mL	0 (0–400)	0 (0–400)	200 (0–800)	0.49
Hospitalization costs, yen	27,820,360 (22,487,728–44,406,513)	44,310,040 (31,899,845–44,502,985)	23,686,340 (20,639,017–25,353,940)	0.20

Data were presented as n (%) for binary variables and median (interquartile range) for continuous variables. ICU-LOS: intensive care unit length of stay, PBD: post-burn day, RBC: Red blood cells, FFP: Fresh frozen plasma, PC: Platelet concentrate.

**Table 3:** Association between NexoBrid use and outcomes.

Outcomes		Estimate	(95% Confidence Interval)	*p*-Value
Non-adjusted				
In-hospital mortality	Odds ratio	0.93	(0.10 to 8.35)	0.95
Bacteremia	Odds ratio	0.28	(0.02 to 4.21)	0.36
ICU-LOS, days	Difference	13	(−25 to 51)	0.51
Total debridement completion, PBD	Difference	−3	(−5 to −1)	0.002
RBC transfusion, mL	Difference	−468	(−2375 to 1439)	0.63
FFP transfusion, mL	Difference	−867	(−3863 to 2128)	0.57
PC transfusion, mL	Difference	1063	(−1045 to 3170)	0.32
Hospitalization costs, yen	Difference	7,629,205	(−5,058,934 to 20,317,345)	0.24
**Propensity score overlap weighting**				
In-hospital mortality	Odds ratio	0.44	(0.03 to 6.14)	0.54
Bacteremia	Odds ratio	0.06	(0.00 to 0.96)	0.047
ICU-LOS, days	Difference	13	(−38 to 64)	0.61
Total debridement completion, PBD	Difference	−4	(−5 to −2)	<0.001
RBC transfusion, mL	Difference	−1918	(−4334 to 498)	0.12
FFP transfusion, mL	Difference	−2872	(−6597 to 853)	0.13
PC transfusion, mL	Difference	1248	(−1720 to 4217)	0.41
Hospitalization costs, yen	Difference	4,565,049	(−9,524,951 to 18,655,049)	0.53
**Propensity score adjusted**				
In-hospital mortality	Odds ratio	0.44	(0.02 to 7.68)	0.57
Bacteremia	Odds ratio	0.02	(0.00 to 0.82)	0.038
ICU-LOS, days	Difference	13	(−36 to 63)	0.60
Total debridement completion, PBD	Difference	−4	(−5 to −2)	<0.001
RBC transfusion, mL	Difference	−1806	(−3921 to 309)	0.09
FFP transfusion, mL	Difference	−2717	(−6217 to 782)	0.13
PC transfusion, mL	Difference	1234	(−1703 to 4172)	0.41
Hospitalization costs, yen	Difference	4,801,067	(−9,125,571 to 18,727,705)	0.50

ICU-LOS: intensive care unit length of stay, PBD: post-burn day, RBC: Red blood cells, FFP: Fresh frozen plasma, PC: Platelet concentrate.

**Table 4:** Details of burn patients with enzymatic debridement.

Patients	Age	TBSA	FTBA	%ED	Date of ED (PBD)	Pre-Soaking	Total Debridement Completion (PBD)	Bacteremia	Outcomes
1	53	44	40	30	2	+	2	+	Survived
2	59	50	35	30	1	+	6	−	Survived
3	21	50	50	30	1	+	2	+	Survived
4	25	95	94	30	1	+	4	+	Dead
5	60	45	32	30	1	+	2	+	Survived
6	54	52	41	15	1	−	2	−	Survived
7	67	55	45	30	1	−	3	+	Dead

TBSA: total burn surface area, FTBA: full-thickness burn surface area, ED: Enzymatic debridement, PBD: post-burn day.

**Table 5:** Details of burn patients with only surgical debridement.

Patients	Age	TBSA	FTBA	Total Debridement Completion (PBD)	Bacteremia	Outcomes
1	55	85	22	7	+	Dead
2	73	58	20	12	+	Dead
3	79	42	28	5	+	Dead
4	38	64	16	6	+	Survived
5	70	45	23	4	+	Survived
6	50	34	18	5	+	Survived
7	80	34	20	3	+	Survived
8	86	32	22	6	+	Survived
9	71	30	0	4	−	Survived
10	49	30	27	7	+	Survived

TBSA: total burn surface area, FTBA: full-thickness burn surface area, PBD: post-burn day.

## Data Availability

The original contributions presented in this study are included in the article. Further inquiries can be directed to the corresponding author.

## Abbreviations

The following abbreviations are used in this manuscript:

TBSA	Total Burn Surface Area
FTBA	Full-Thickness Burn surface Area
RBC	Red Blood Cells
FFP	Fresh Frozen Plasma
PC	Platelet Concentrate
